# Walking the Talk for Dementia 2024: Building Social Networks and Collaborations

**DOI:** 10.1002/alz70858_097740

**Published:** 2025-12-24

**Authors:** Sherril B Gelmon, Jenn Hollandsworth Reed, Jonathan Adrian Zegarra‐Valdivia, Walter D Dawson, Lindsey Smith, Lingani Mbakile‐Mahlanza, Kate Irving, Fernando Aguzzoli Peres

**Affiliations:** ^1^ OHSU‐PSU School of Public Health, Portland, OR, USA; ^2^ GBHI Lived Experience Panel, Portland, OR, USA; ^3^ Achucarro Basque Center for Neuroscience, Leioa, Spain; ^4^ Global Brain Health Institute (GBHI), University of California San Francisco, San Francisco, CA, USA; ^5^ Universidad Señor de Sipán, Chiclayo, Peru; ^6^ Global Brain Health Institute, San Francisco, CA, USA; ^7^ Oregon Health & Science University, Portland, OR, USA; ^8^ Portland State University, Portland, OR, USA; ^9^ OHSU‐PSU School of Public Health, Portland, OR, Portland, OR, USA; ^10^ University of Botswana, Gaborone, Botswana; ^11^ Dublin City University, Dublin, Ireland; ^12^ Global Brain Health Institute, Dublin, Dublin, Ireland; ^13^ Walking the Talk for Dementia, Porto Alegre, Rio Grande do Sul, Brazil

## Abstract

**Background:**

Walking the Talk for Dementia (WTD) 2024 encouraged multi‐stakeholder collaborations in dementia research, care, and advocacy. The weeklong event offered many opportunities to build social networks and collaborations through walking 40 km. along the Camino de Santiago de Compostela in northern Spain, the formal symposium, and multiple unstructured events.

**Methods:**

Data collection documented pre‐existing and newly established connections and collaborations using social network analysis (SNA), an effective technique for characterizing and visualizing social networks. Data were collected through pre‐ and post‐surveys administered using Qualtrics. Survey questions assessed the kind of connections each participant had with other WTD participants – knowing a participant, having a personal connection, planning to collaborate in future, and currently collaborating. Responses were used to visualize social networks before and after WTD and calculate centrality (number of connections), closeness (distances between participants), and betweenness (bridges between participant clusters). Understanding of social networks was further informed by survey questions related to collaborations, satisfaction, inspiration, and written, audio and video personal reflections.

**Results:**

Sixty‐two participants completed the pre‐ and post‐surveys social network questions (87.3% and 80.1% of study participants, respectively). Preliminary analyses identify certain individuals as central players in the WTD social network. Further analyses by participant role (clinician, person with dementia, advocate, etc.) and geographic region reveal that networks transcend boundaries of role and geography.

Social network visualizations will be augmented by personal reflections and survey responses. Participants identified connections, new collaborations, and plans for future collaborations through WTD. These focus on advocacy, education, research, and inter‐organizational partnerships. Key themes included a commitment to meeting people and deliberate engagement during WTD, and plans for the integration in the future of people with lived experience in research, education, advocacy, and policy work.

**Conclusions:**

Many participants demonstrated active or planned collaborations with others in the WTD community. This research demonstrates that WTD achieved its goal of fostering multi‐stakeholder collaborations in dementia research, care, and advocacy. Future plans include another survey to document the evolution of the networks, adding WTD 2023 participants to demonstrate the growing connections among WTD communities.

